# Enhancing Exosome Production from Adipose-Derived Stem Cells via Arachidonic Acid Priming for Diabetic Wound Healing

**DOI:** 10.3390/ijms27209029

**Published:** 2026-10-12

**Authors:** Yujiao Cai, Jinnan Wang, Ruobing Zhang, Dehui Che, Juan Xie, Dongsheng Cao

**Affiliations:** Department of Plastic and Reconstructive, The Second Affiliated Hospital of Anhui Medical University, Hefei 230601, China; 15256205220@163.com (Y.C.);

**Keywords:** adipose-derived mesenchymal stem cells, exosomes, diabetic wound, angiogenesis, exocytosis, arachidonic acid

## Abstract

Adipose-derived stem cell (ADSC)-derived exosomes are promising for regenerative therapy. However, their clinical translation is hindered by limited secretion yield and reduced functional potency. Here, we tested whether arachidonic acid (AA) priming increases exosome production while preserving exosomal identity, and whether AA-primed exosomes (AA-Exos) enhance diabetic wound repair. ADSCs were treated with AA, and exosome yield was quantified using particle counts and cumulative acetylcholinesterase (AChE) activity. Exosome morphology and canonical markers were assessed to confirm identity. Mechanistically, transcriptomic and functional analyses revealed that AA promoted multivesicular body and intraluminal vesicle formation through CHMP1B upregulation and facilitated exosome release through Rab27b, and knockdown of either gene impaired the corresponding process. Proteomic profiling further showed that AA-Exos were enriched in wound-repair-related cargo, particularly interleukin-8 (IL-8/CXCL8). In high-glucose-challenged endothelial cells, AA-Exos enhanced proliferation and migration and reduced apoptosis by activating FAK/AKT/ERK signaling, whereas IL-8 neutralization attenuated these effects. In a diabetic mouse model, AA-Exos accelerated wound closure and improved tissue perfusion, angiogenesis, and collagen deposition. Taken together, AA priming enhances exosome production while improving their therapeutic function, suggesting a practical strategy to strengthen the translational potential of ADSC-derived exosomes for diabetic wound regeneration.

## 1. Introduction

In the field of regenerative therapy, stem cell-based technologies have achieved rapid development, and human mesenchymal stem cells (MSCs) stand out as the most widely studied seed cells for clinical translational research [1,2,3]. Accumulating evidence has demonstrated that the therapeutic effects of MSCs are mainly dependent on their paracrine secretion function, rather than direct cell differentiation, and MSC-derived exosomes (MSC-Exos) serve as the primary carrier of MSC paracrine bioactive components [4]. Extracellular exosomes are nanoscale vesicles with diameters ranging from 30 to 150 nm, which are secreted by viable cells following the fusion of intracellular multivesicular bodies with the cell plasma membrane. As crucial intercellular signaling mediators, these vesicles participate in a wide array of physiological and pathological biological processes in organisms [5,6,7]. Compared with traditional intact stem cell transplantation, MSC-Exos-based cell-free therapy exhibits multiple irreplaceable advantages: it effectively avoids the tumorigenic risk of stem cell infusion, features lower immune reactivity, reduces biological contamination risks, and allows convenient long-term storage and transportation [8,9,10]. For these reasons, MSC-Exos have been recognized as a superior alternative to conventional stem cell therapy and possess great clinical application potential as novel advanced therapy medicinal products (ATMPs) [11]. A large number of preclinical studies have verified the broad therapeutic prospects of MSC-Exos in multiple tissue injury repair and disease intervention. Mechanistically, bone marrow MSC-Exos can be internalized by adipocytes, myocytes and hepatocytes, and further regulate insulin sensitivity both in vitro and in vivo, providing new insights into metabolic disease research [12]. In orthopedic tissue repair, MSC-Exos can promote chondrocyte proliferation and infiltration, modulate local immune microenvironment, suppress chondrocyte apoptosis, and enhance the synthesis of type II and type VI collagen in injured sites, thus efficiently repairing osteochondral tissue defects [13].

Despite the promising application prospects of MSC-Exos, multiple bottlenecks still restrict their large-scale clinical translation and commercialization. First, current isolation and purification strategies for exosomes suffer from relatively low efficiency, resulting in unsatisfactory input-output ratios in industrial and clinical production [14,15,16]. Second, the biogenesis, cargo loading and secretion of MSC-Exos are susceptible to multiple extrinsic and intrinsic factors, including cell types, culture conditions, cell viability and passage number [17]. Such variability inevitably leads to inconsistent quality and unstable therapeutic efficacy of exosome products across different batches. To further improve the therapeutic performance of MSC-Exos and accelerate their clinical transformation, it is urgent to develop advanced strategies to enhance exosome yield per single cell and optimize exosome cargo packaging, which is essential for standardized and high-efficiency exosome-based therapy [18,19]. Therefore, in addition to improving and optimizing exosome isolation methods, enhancing cells’ exosome-producing capacity by directly modifying cells or regulating the cellular microenvironment represents another strategy to obtain sufficient exosomes for regenerative medicine. Our previous studies had also demonstrated that AA-Exos had better therapeutic effects on diabetic wounds than AA or Exos alone [20]. However, the study did not examine the exosomes themselves nor the cargoes carried by the exosomes. Our current study reveals novel insights into how the proteins loaded into these exosomes influence HUVEC proliferation and wound healing. The key finding is that AA priming significantly increases exosome secretion yield from ADSCs, providing a practical strategy to enhance exosome production for further applications.

Multiple biotechnological techniques have been exploited to modulate the exosome production capacity of donor cells [21,22]. Functionally, nSMases mediate the hydrolysis of sphingomyelin to produce ceramide. The accumulated ceramide further induces the fusion of small membrane microdomains into larger membrane domains and triggers domain-dependent membrane budding, thereby initiating the formation of exosomal vesicles [23,24]. Wu et al. reported that Bioglass^®^ ionic products increased MSC exosome production by upregulating nSMase2 and Rab27a, thereby activating the nSMase and Rab GTPase pathways, respectively. They also found reduced miR-342-5p and elevated miR-1290 levels in exosomes, which enhanced endothelial cell angiogenesis and dermal neovascularization in vivo [25]. Wang et al. found that the Wnt agonist CHIR99021 increased hucMSC exosome production, partly through SNAP25-mediated exocytosis, and upregulated proteins involved in migration and wound healing. In diabetic mice, CHIR-Exos accelerated wound closure, promoted collagen deposition and angiogenesis, and attenuated chronic inflammation [26].

In our study, arachidonic acid pretreatment effectively increased the exosome secretion rate of adipose-derived stem cells (ADSCs). ADSCs are abundant and possess high cellular viability, making them widely utilized in stem cell-based therapeutic applications. We also evaluated the biological effects of the resulting exosomes. Nanoparticle tracking analysis (NTA) was used to determine the size distribution and concentration of secreted exosome particles. ADSCs transcriptomic analysis and exosomal proteomics were performed to identify enriched signaling pathways and enhanced functional properties. Based on the proteomic findings, appropriate animal models and functional validation experiments were selected and conducted. Through this work, we established a safe, stable, and effective pretreatment strategy to increase exosome production and improve therapeutic efficacy, thereby supporting the clinical translation and commercialization of ADSC-derived exosomes (ADSC-Exos).

## 2. Results

### 2.1. AA Pretreatment Preserves ADSCs and Exosome Properties

**Figure 1:**
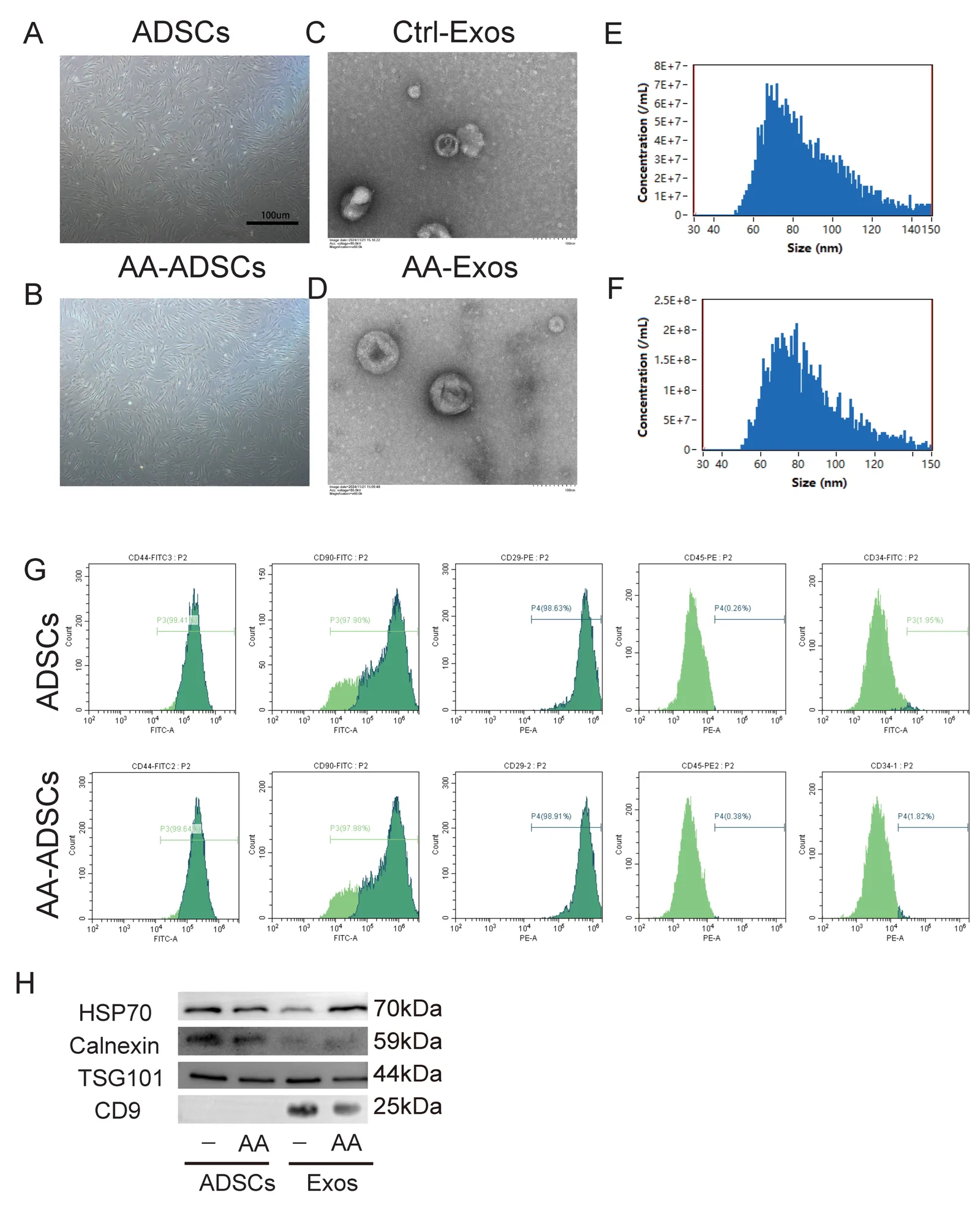
Characterization of AA-preconditioned ADSCs and derived exosomes. (**A**,**B**) Morphology of untreated ADSCs and AA-pretreated ADSCs. (**C**,**D**) TEM micrographs of Ctrl-Exos and AA-Exos. (**E**,**F**) Size distribution profiles of Ctrl-Exos and AA-Exos. (**G**) Flow-cytometric analysis of surface marker expression for untreated and AA-treated ADSCs. (**H**) Western blot detection of exosome-associated markers in ADSCs and isolated exosomes.

AA pretreatment induced no obvious morphological alterations in ADSCs relative to untreated counterparts (Figure 1A,B). Furthermore, flow cytometry analysis revealed that differentially treated ADSCs exhibited comparable positive expression levels of CD44, CD90, CD29, as well as consistent negative expression of CD45 and CD34 (Figure 1G). These data demonstrated that the AA treatment did not substantially modify the surface marker profile of ADSCs. Images acquired via transmission electron microscopy (TEM) and high-sensitivity flow cytometry (HSFCM) clearly demonstrated that AA-preconditioned ADSC-secreted exosomes (AA-Exos) and exosomes harvested from conventionally cultured ADSCs (Ctrl-Exos) displayed characteristic round or cup-shaped membranous vesicles (Figure 1C,D), with a size distribution spanning 50–120 nm and a mean particle size of 70 ± 5 nm (Figure 1E,F). Furthermore, flow cytometry analysis demonstrated that ADSCs subjected to different treatments exhibited comparable levels of negative CD34 and CD45 expression, as well as positive CD44, CD90, and CD29 expression, suggesting that these treatments did not significantly alter the surface marker profile of ADSCs (Figure 1G). Western blotting confirmed abundant enrichment of canonical exosomal marker proteins, namely CD9, Hsp70, and Tsg101, in both AA-Exos and Ctrl-Exos preparations (Figure 1H). By contrast, Calnexin—a resident endoplasmic reticulum protein absent from exosomal cargo—was scarcely detected in either experimental group. Collectively, the isolated vesicles were verified as bona fide exosomes; moreover, AA-Exos and Ctrl-Exos showed no statistically distinguishable disparities in vesicle morphology, size distribution, or marker protein abundance.

### 2.2. AA Pretreatment Enhances ADSC-Derived Exosome Production

**Figure 2:**
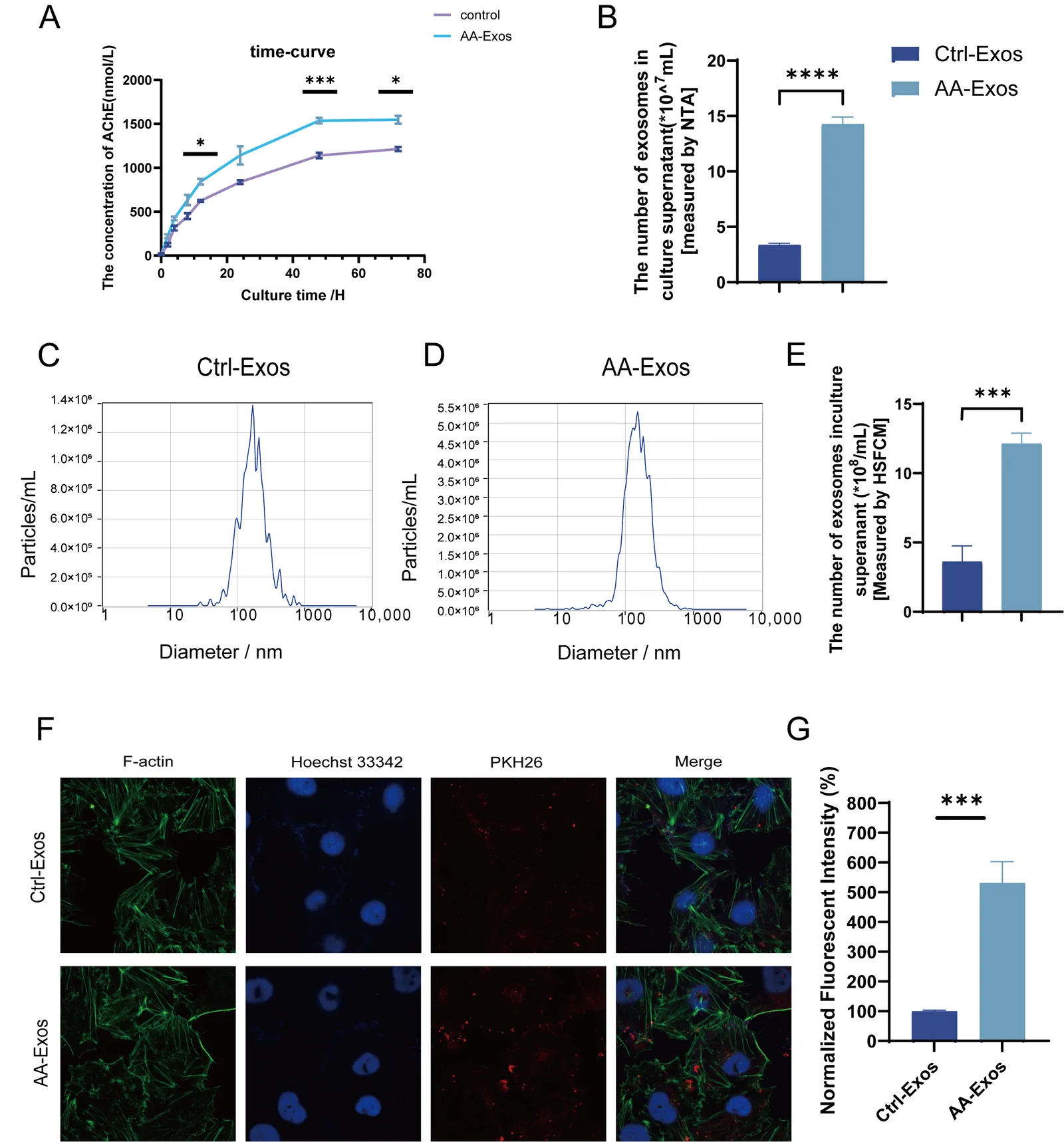
AA enhances ADSCs-derived exosome secretion and exosome uptake in HUVECs. (**A**) Time-dependent AChE concentration curves of Ctrl-Exos and AA-Exos. (**B**) NTA quantification of exosome particle abundance in culture supernatants. (**C**,**D**) NTA-based particle-size distribution of Ctrl-Exos and AA-Exos. (**E**) Exosome particle counts measured by HSFCM. (**F**) Representative fluorescence micrographs showing PKH26-labelled exosome internalization in HUVECs. (Objective (63×)) (**G**) Quantitative analysis of normalized intracellular red fluorescence intensity for exosome internalization. The three bar graphs (Panel (**B**,**E**,**G**)) in Figure 2 share exactly the same experimental groups (Ctrl-Exos and AA-Exos). Data are mean ± SD. * *p* < 0.05, *** *p* < 0.001, **** *p* < 0.0001.

We investigated the potential effects of arachidonic acid on exosome secretion from ADSCs. Prior studies have demonstrated that exosome abundance in culture supernatants is positively correlated with acetylcholinesterase (AChE) concentrations [25]. Accordingly, we first quantified AChE levels in ADSCs culture supernatants as an indicator of exosome yield. To ensure accurate comparisons across groups, we normalized exosome abundance to the total cellular protein content of each sample. Figure 2A–E illustrate how AA regulates exosome secretion from ADSCs. We quantified exosome yields by measuring supernatant AChE levels and particle counts via multiple analytical platforms. As shown in Figure 2A, supernatant AChE levels accumulated progressively in both groups over time. No significant intergroup difference was detected within the initial 12 h. After 12 h, cumulative AChE levels in the AA-Exos group rose steadily and remained markedly higher than the Ctrl-Exos group throughout the subsequent 60 h. To further compare exosome abundance between groups, we quantified exosome concentrations in ADSCs culture supernatants collected at the 48 h time point using NTA and HSFCM assays. These data verified that the AA-Exos group contained substantially more exosome particles than the control group (Figure 2B–E). We further validated cellular uptake of exosomes by human umbilical vein endothelial cells (HUVECs). PKH26-labelled exosomes emit red fluorescent signals upon 561 nm laser excitation. We incubated HUVECs with AA-Exos or Ctrl-Exos at a fixed concentration of 50 μg/mL for 24 h, and distinct red fluorescent exosome puncta were visualized inside HUVECs (Figure 2F). Quantitative analysis of intracellular red fluorescence intensity revealed that AA-Exos showed higher intracellular uptake than Ctrl-Exos at the 24 h time point under identical incubation conditions (Figure 2G).

### 2.3. AA Promotes Exosome Secretion by Upregulating Rab27b and CHMP1B Expression

**Figure 3:**
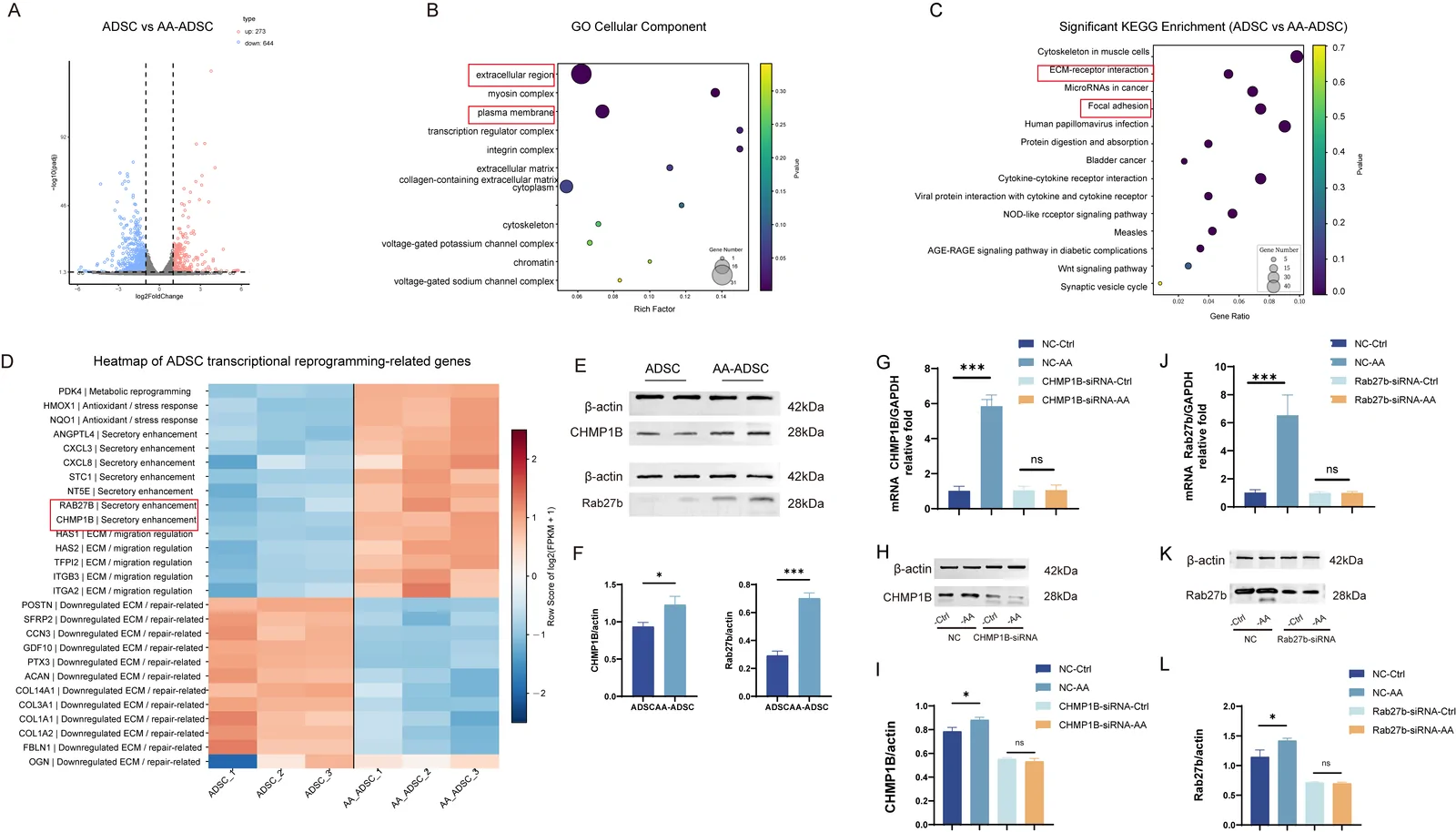
Transcriptomic analysis reveals CHMP1B and RAB27B mediate AA-enhanced secretion of ADSCs. (**A**) Volcano plot illustrating differentially expressed genes (DEGs) in ADSCs with versus without AA treatment. (**B**) GO cellular-component enrichment of DEGs, highlighting enrichment in extracellular region and plasma membrane. (**C**) KEGG enrichment analysis showing enriched ECM-receptor interaction and Focal adhesion pathways. (**D**) Heatmap of DEGs linked to metabolic reprogramming, antioxidant-stress response, secretion, ECM and migration regulation; CHMP1B and RAB27B are up-regulated after AA exposure. (**E**,**F**) Western blot and quantitative analysis showing elevated CHMP1B and RAB27B protein in AA-treated ADSCs. β-actin s loading control. (**G**,**J**) qPCR detection of CHMP1B and RAB27B mRNA after si-NC/siRNA transfection in the presence or absence of AA. (**H**,**I**,**K**,**L**) Western blot representative images and quantitative analysis of CHMP1B and RAB27B protein upon siRNA-mediated knockdown, with or without AA stimulation. Data are mean ± SD. * *p* < 0.05, *** *p* < 0.001; ns, not significant.

Accumulating evidence indicates that exosome biogenesis is mainly controlled by two core processes: biogenesis of multivesicular bodies-derived intraluminal vesicles and subsequent exocytosis. To explore the mechanism underlying enhanced secretion, we performed transcriptomic analysis on ADSCs treated with or without AA. Volcano plot analysis identified differentially up- and down-regulated genes (Figure 3A). Subsequent Gene Ontology (GO) analysis showed enrichment of differentially expressed genes in two terms: extracellular region and plasma membrane (Figure 3B). Furthermore, KEGG analysis revealed enrichment for the ECM-receptor interaction and Focal adhesion pathways. These findings indicate that AA pretreatment modulates exosome secretion and release (Figure 3C). Cluster heatmap analysis of transcriptomic differentially expressed genes demonstrated that AA pretreatment drove ADSCs toward a phenotype characterized by metabolic reprogramming, antioxidant-stress responses, enhanced secretory capacity, as well as ECM and migration-related regulation, as shown in the corresponding heatmap. Notably, CHMP1B and RAB27B were among the up-regulated genes (Figure 3D). CHMP1B mediates intraluminal membrane cleavage during ILV generation for MVB biogenesis, whereas RAB27B is critical for vesicle exocytosis (Figure 3E,F).

We next performed Western blotting to validate these observations. AA treatment induced elevated expression of CHMP1B and RAB27B. Si-NC served as the negative control, and ADSCs were transfected with CHMP1B-siRNA or RAB27B-siRNA. As presented in Figure 3G–I, 48 h of AA stimulation increased CHMP1B transcript levels compared with the si-NC group, and protein expression was significantly enhanced at 72 h. Similarly, AA treatment up-regulated RAB27B mRNA at 48 h, accompanied by significantly increased RAB27B protein expression at 72 h (Figure 3J–L). Upon CHMP1B-siRNA and RAB27B-siRNA transfection, no significant differences in CHMP1B and RAB27B mRNA levels, as well as their corresponding protein expression, were observed between si-NC-control and AA-treated ADSCs.

**Figure 4:**
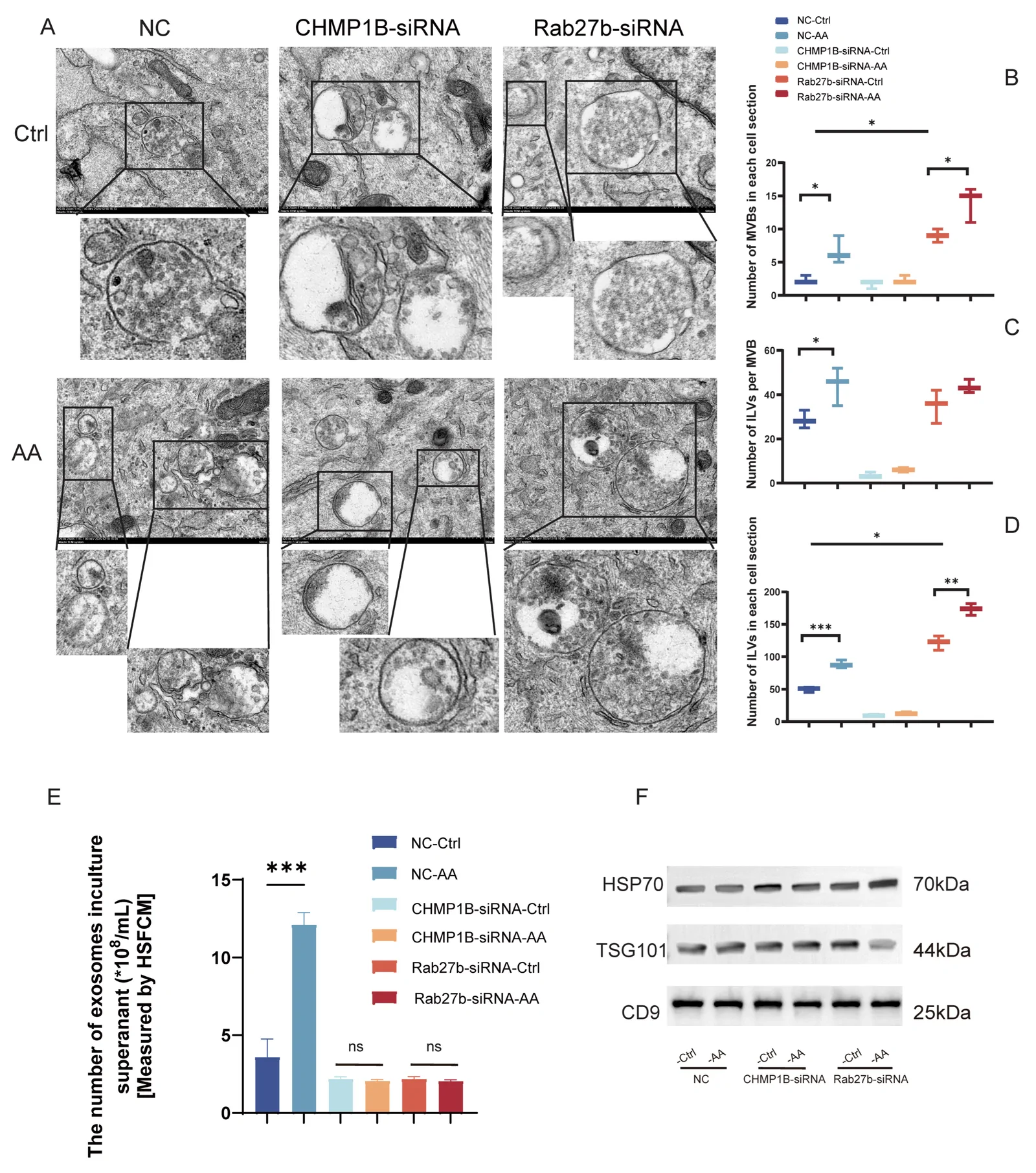
Effects of CHMP1B and RAB27B knockdown on AA-promoted exosome biogenesis and release in ADSCs. (**A**) Representative TEM images of intracellular MVBs and ILVs in ADSCs transfected with si-NC, CHMP1B-siRNA or Rab27b-siRNA, with or without AA treatment. Magnified views of boxed regions are shown below each panel. (**B**) Quantification of MVB number per cell section. (**C**) Quantification of ILV number per single MVB. (**D**) Quantification of total ILV number per cell section. (**E**) Exosome particle concentration in culture supernatant, measured by HSFCM. (**F**) Western blot detection of exosomal markers HSP70, TSG101 and CD9 in isolated exosomes. Data are mean ± SD. * *p* < 0.05, ** *p* < 0.01, *** *p* < 0.001; ns, not significant.

To further elucidate the mechanism underlying AA-enhanced exosome secretion in ADSCs, transmission electron microscopy (TEM) was performed to detect and quantify multivesicular bodies (MVBs) and intraluminal vesicles (ILVs) in the cytoplasm. As shown in Figure 4A, typical MVB structures enclosing abundant ILVs were clearly observed in the cytoplasm of all groups. Quantitative statistics were calculated based on images from over 15 individual cells per group. As presented in Figure 4B, the number of MVBs per cell section in the NC-AA group was significantly higher than that in the NC-Ctrl group (*p* < 0.05). CHMP1B knockdown markedly abrogated the AA-induced increase in MVB abundance, and no significant difference was found between the CHMP1B-siRNA-Ctrl and CHMP1B-siRNA-AA groups. In contrast, Rab27b silencing led to intracellular accumulation of MVBs. The number of MVBs per cell section in both Rab27b-siRNA groups was higher than that in the NC-Ctrl group, and AA treatment further elevated MVB number in Rab27b-knockdown ADSCs (*p* < 0.05). In addition, the number of ILVs within each single MVB was counted, and the results are shown in Figure 4C. AA stimulation significantly increased ILV number per MVB in NC-transfected ADSCs compared with the NC-Ctrl group (*p* < 0.05). CHMP1B silencing drastically reduced ILV content inside MVBs regardless of AA treatment, indicating impaired ILV biogenesis. For Rab27b-siRNA groups, ILV number per MVB showed no obvious difference from the NC-Ctrl group, and AA treatment still induced a moderate increase. Furthermore, quantitative results of total ILV number per cell section are displayed in Figure 4D. Compared with the NC-Ctrl group, the NC-AA group exhibited a remarkably increased total ILV number per cell section (*p* < 0.001). CHMP1B knockdown nearly abolished the AA-promoted elevation of total ILVs, with both CHMP1B-siRNA groups showing very low ILV counts. Consistent with MVB accumulation, Rab27b-silenced ADSCs had significantly more total ILVs per cell than NC-Ctrl cells, and AA treatment further augmented this effect (*p* < 0.01).

In addition, exosome particles in the culture supernatant were quantified by high-sensitivity flow cytometry (HSFCM). After ADSCs were treated with CHMP1B-siRNA or Rab27b-siRNA, the numbers of exosome particles in the supernatant of siRNA-transfected ADSCs cultured with or without AA were markedly decreased, and there was no significant difference between the siRNA-Ctrl and siRNA-AA groups (Figure 4E). Noteworthily, the exosome concentrations of CHMP1B- or Rab27b-silenced ADSCs were hardly enhanced even when the cells were stimulated with AA, while a significant increase was observed in NC-transfected ADSCs upon AA treatment (*p* < 0.001). Furthermore, Western blot analysis of the isolated exosomes confirmed positive expression of the exosomal markers CD9, HSP70, and TSG101 (Figure 4F). These results confirm that all the above treatments do not significantly alter the fundamental characteristics of exosomes.

Collectively, AA upregulates the mRNA and protein expression of CHMP1B and RAB27B, which promotes intracellular exosome biogenesis and activates Rab GTPase signaling, thus facilitating the release of mature exosomes.

### 2.4. AA Facilitates the Loading of Wound-Healing-Related Proteins into Exosomes

**Figure 5:**
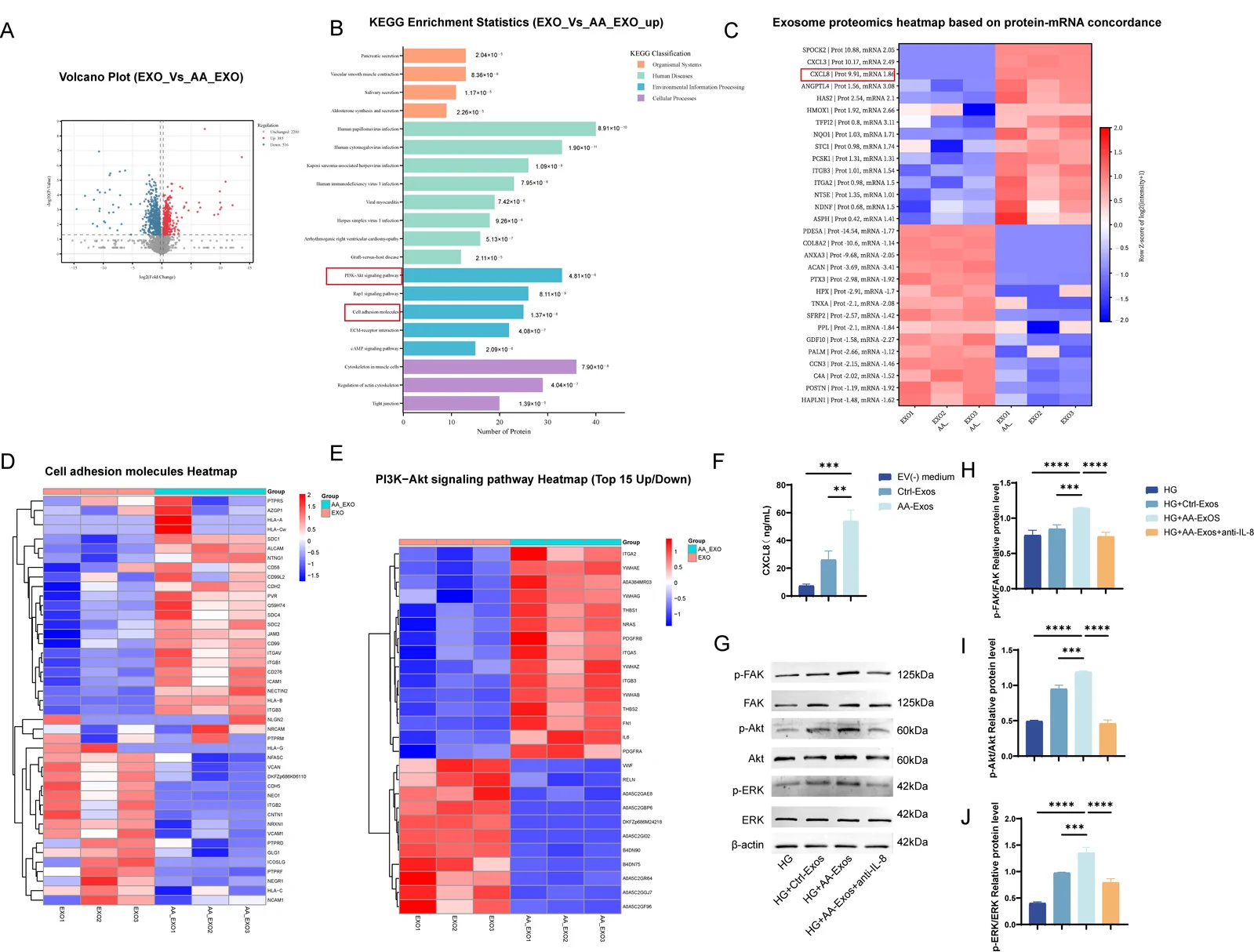
Proteomic and mechanistic analysis of AA-primed ADSC-derived exosomes. (**A**) Volcano plot of differentially expressed proteins between Ctrl-Exos and AA-Exos. (**B**) KEGG pathway enrichment of upregulated proteins in AA-Exos. (**C**) Heatmap of protein-mRNA concordance for candidate wound-healing molecules. (**D**,**E**) Clustering heatmaps of differential cell adhesion molecules (**D**) and PI3K-Akt pathway proteins (**E**). (**F**) IL-8 concentrations in EV-depleted medium, Ctrl-Exos and AA-Exos measured by ELISA. (**G**) Representative Western blots of FAK, Akt and ERK phosphorylation under control and anti-IL-8 neutralization conditions. (**H**–**J**) Densitometric quantification of p-FAK/FAK (**H**), p-Akt/Akt (**I**) and p-ERK/ERK (**J**) ratios. Data are mean ± SD. ** *p* < 0.01, *** *p* < 0.001, **** *p* < 0.0001.

As shown in Figure 5, AA significantly enhanced exosome secretion-associated protein pathways mediated by CHMP1B and RAB27B in ADSCs. However, increased exosome production does not necessarily equate to improved therapeutic efficacy. Therefore, we performed proteomic analysis on exosomes derived from AA-ADSCs to investigate their cargo remodeling effects. The volcano plot (Figure 5A) revealed a distinct profile of differentially expressed proteins between AA-Exos and Ctrl-Exos, with both upregulated and downregulated proteins identified. KEGG enrichment analysis (Figure 5B) indicated that these differentially expressed proteins were mainly enriched in two pathways: the PI3K–Akt signaling pathway and cell adhesion molecules. Further clustering heatmaps (Figure 5D,E) demonstrated that multiple adhesion and receptor signaling molecules highly relevant to wound healing and cell migration were upregulated within these pathways, including ALCAM, ICAM1/VCAM1, the integrin (ITG) axis (ITGB1 and ITGA-related molecules), NCAM1, PDGFRB/PDGFRA, NRAS. To verify the concordance between transcriptional and translational changes, we integrated the expression trends from the ADSC transcriptome and exosomal proteome, and found that key candidate molecules including CXCL8 (IL-8), ANGPTL4, HAS2, ITGA2, and ITGB3 were significantly enriched and upregulated at the exosomal level (Figure 5C). Subsequent IL-8 ELISA using exosome lysates showed that the IL-8 concentration in AA-Exos was significantly higher than that in control Exos, and a clear difference was also observed compared with Exo-depleted culture supernatant (Figure 5F). Mechanistically, the pro-migratory downstream signaling targets of IL-8—including p-FAK/FAK, p-AKT/Akt, and p-ERK/ERK—were significantly elevated after AA-Exos treatment; administration of anti-IL-8 neutralizing antibody markedly suppressed these phosphorylation-to-total protein ratios (Figure 5G–J). Collectively, AA not only increases exosome release but also remodels exosomal cargo (especially IL-8 and adhesion/ECM-related factors) to activate the IL-8–FAK/AKT/ERK signaling network, providing a molecular basis for AA-mediated promotion of wound healing and cell migration.

### 2.5. AA-Exos Exhibited a Superior Pro-Healing Effect Compared with Ctrl-Exos In Vivo

**Figure 6:**
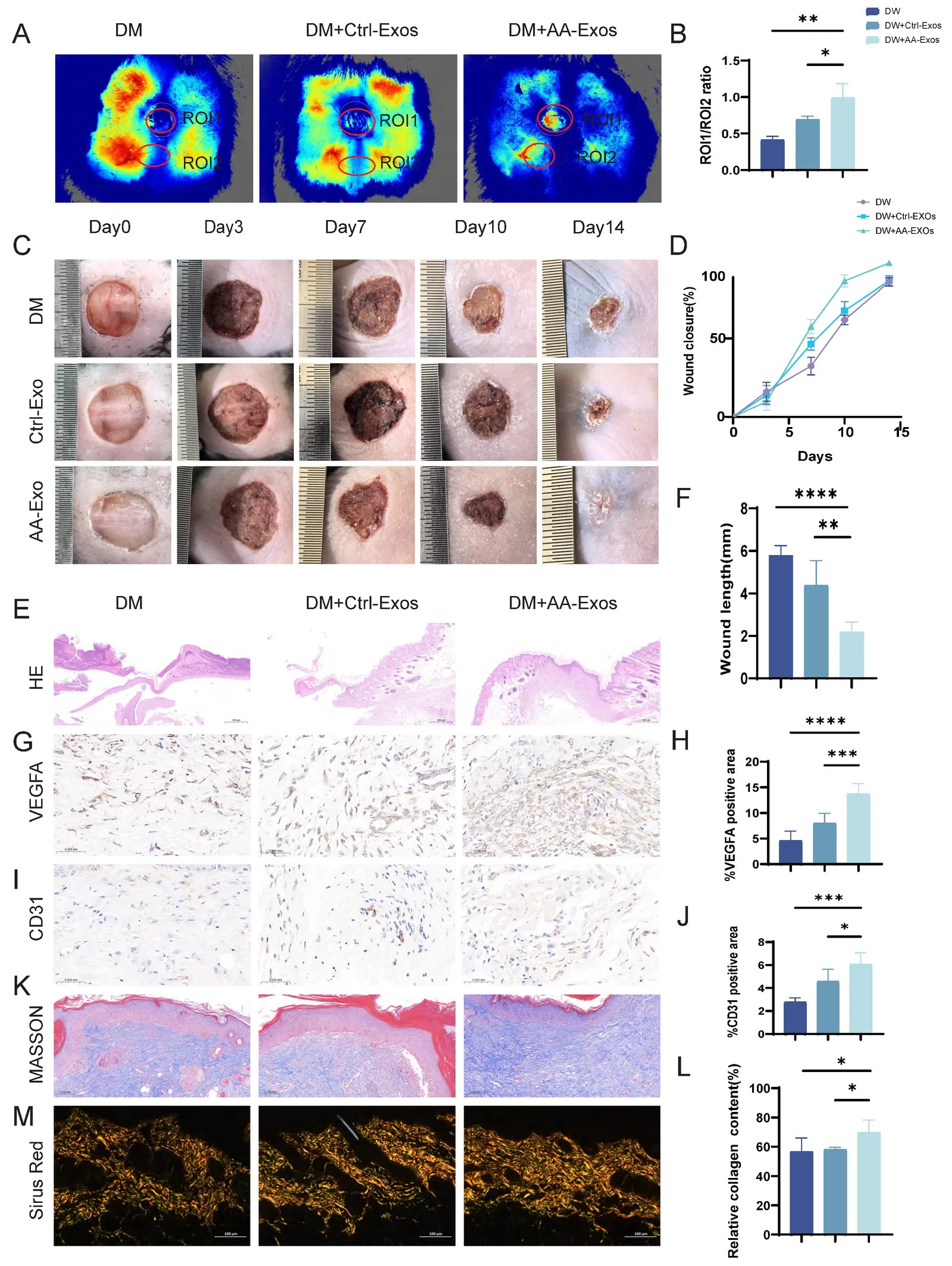
AA-Exos accelerate diabetic wound healing and enhance angiogenesis and collagen remodeling in vivo. (**A**,**B**) Representative images of laser speckle imaging on day 10 and quantitative analysis of perfusion ratio (ROI1/ROI2) in diabetic wounds. (**C**,**D**) Representative macroscopic wound images over time and the corresponding wound closure/size changes. (**E**) H&E staining of wound tissues on day 14. (**F**) Quantification of wound length during the healing process. (**G**–**J**) Immunohistochemical staining and quantification of VEGFA (**G**,**H**) and CD31 (**I**,**J**) in wound tissues on day 14. (**K**–**M**) Masson staining and Sirius Red staining (**K**) and quantitative analysis of collagen deposition (**L**,**M**) in wound beds on day 14. Data are mean ± SD. * *p* < 0.05, ** *p* < 0.01, *** *p* < 0.001, **** *p* < 0.0001.

We established a full-thickness skin wound model in db/db type 2 diabetes mellitus mice and monitored wound closure at days 0, 3, 7, 10, and 14. Representative images showed that AA-Exos markedly accelerated wound repair, with wounds in the AA-Exos group approaching near-complete closure by day 14, whereas the DM (diabetes mellitus) and DM + Ctrl-Exos groups remained incompletely healed (Figure 6C,D). Consistently, quantitative analysis of wound length further confirmed the enhanced wound closure efficiency of AA-Exos (Figure 6F). To evaluate restoration of local blood perfusion, laser speckle imaging was performed on day 10. The perfusion ratio between the wound center and surrounding normal skin (ROI1/ROI2) was significantly higher in the AA-Exos group than in the DM and DM + Ctrl-Exos groups, indicating improved microcirculation recovery (Figure 6A,B). Histological examination on day 14 demonstrated improved tissue regeneration in the AA-Exos group. H&E staining revealed better re-epithelialization and more organized tissue architecture relative to the control groups (Figure 6E). Masson’s staining and Sirius Red staining further showed increased collagen deposition and more orderly collagen fiber arrangement in AA-Exos-treated wounds (Figure 6K–M). Immunohistochemical analyses indicated that AA-Exos significantly upregulated vascular-related markers, with higher expression of VEGFA and CD31, suggesting enhanced angiogenesis (Figure 6G–J). Collectively, these findings demonstrate that AA-Exos promote diabetic wound healing by improving blood perfusion/angiogenesis, enhancing collagen deposition, and improving collagen organization.

### 2.6. AA-Exos Promote HUVEC Migration and Proliferation While Inhibiting Apoptosis via IL-8

**Figure 7:**
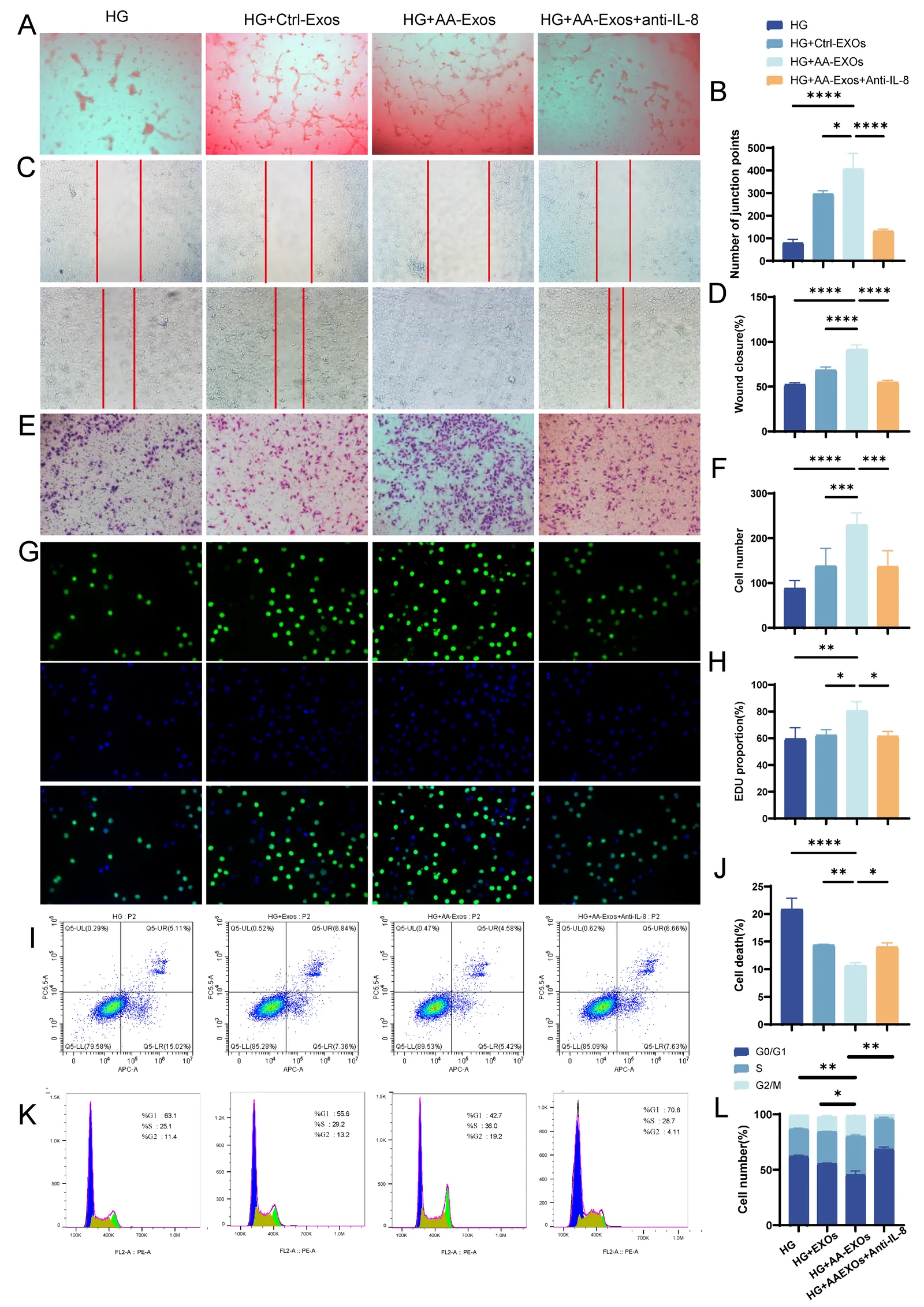
AA-Exos promotes HG-induced endothelial functional recovery via IL-8–dependent mechanisms. Panels (**A**,**C**,**E**,**G**) in the figure were captured at a magnification of ×100. (**A**) Representative micrographs of capillary-like tube formation on Matrigel. (**B**) Quantitative analysis of the number of tube branch junctions. (**C**) Representative phase-contrast images of the scratch wound-healing assay at 0 h (upper row) and 48 h (lower row); red lines denote the initial wound boundaries. (**D**) Quantification of wound closure (%). (**E**) Representative crystal violet–stained images of Transwell migration. (**F**) Quantification of migrated cell numbers. (**G**) Representative immunofluorescence images of EdU incorporation (green, upper row), DAPI nuclear counterstaining (blue, middle row), and merged channels (lower row). (**H**) Quantification of the percentage of EdU-positive cells. (**I**) Representative flow cytometry dot plots of Annexin V/PI double-stained cells for apoptosis assessment. (**J**) Quantification of the percentage of dead cells. (**K**) Representative flow cytometry histograms of cell-cycle distribution (PI staining). (**L**) Quantification of the relative proportions of cells in G0/G1, S, and G2/M phases. Data are mean ± SD. * *p* < 0.05, ** *p* < 0.01, *** *p* < 0.001, **** *p* < 0.0001.

In in vitro experiments, HUVECs were divided into four treatment groups: the HG group (treated with high-glucose culture medium only), the HG + Ctrl-Exos group, the HG + AA-Exos group, and the HG + AA-Exos + anti–IL-8 group (plus IL-8 neutralizing antibody). The results indicate that AA-Exos significantly promotes endothelial repair in HG-stimulated cells. In the angiogenesis-related assay, AA-Exos-treated cells exhibited markedly enhanced tube formation compared with the HG and HG + Ctrl-Exos groups, as reflected by increased tube formation (Figure 7A) and a higher number of tube branch junctions (Figure 7B). Importantly, blockade of IL-8 substantially weakened the pro-angiogenic effects of AA-Exos (Figure 7A,B). In the migration assay, the scratch wound-healing experiment showed that AA-Exos accelerated wound closure and increased wound-healing coverage relative to HG and HG + Ctrl-Exos (Figure 7C,D). Consistently, IL-8 neutralization significantly attenuated the pro-migratory effect of AA-Exos (Figure 7C,D). In the transwell assay, AA-Exos increased the number of cells that migrated across the membrane. Moreover, anti–IL-8 treatment significantly inhibited the migration enhancement induced by AA-Exos (Figure 7E,F). With respect to proliferation, EdU staining and quantification demonstrated that AA-Exos increased the number and proportion of EdU-positive cells under HG conditions, indicating enhanced proliferative activity. After IL-8 blockade, the AA-Exos–mediated pro-proliferative effect was significantly reduced (Figure 7G,H). Apoptosis analysis further showed that Annexin V/PI staining revealed a decrease in HG-induced apoptotic cell levels in the AA-Exos group (Figure 7I,J). Notably, anti–IL-8 markedly reversed the anti-apoptotic effect of AA-Exos (Figure 7I,J). Finally, flow cytometry analysis of the cell cycle showed that AA-Exos promoted cell-cycle progression by increasing the percentages of S-phase and G2/M-phase cells compared with the HG control. This cell-cycle–promoting effect was substantially attenuated by anti–IL-8 treatment (Figure 7K,L). In summary, AA-Exos promotes HG-induced endothelial functional recovery by enhancing angiogenesis and migration, stimulating proliferation, and inhibiting apoptosis. These beneficial effects are partly dependent on IL-8 signaling.

## 3. Discussion

Exosome-based therapies offer a cell-free alternative for diabetic wound repair, combining potent paracrine bioactivity with reduced risks associated with live-cell administration. Despite extensive preclinical progress, clinical translation is constrained by two closely linked issues: (i) limited exosome yield for scalable manufacturing and (ii) variability in exosome cargo composition that drives inconsistent therapeutic efficacy across batches and donor conditions [5,16,17]. In this study, we addressed these bottlenecks using a donor-cell priming strategy with arachidonic acid (AA) to enhance exosome production from ADSCs while preserving core exosome identity. Mechanistically, AA increased MVB/ILV biogenesis and exocytosis regulators, particularly CHMP1B and Rab27b, thereby strengthening the secretion pathway. Functionally, AA-primed exosomes remodeled the exosomal proteome toward wound-repair–relevant programs, with IL-8 (CXCL8) acting as a key mediator that activates downstream IL-8–FAK/AKT/ERK signaling in endothelial cells. Accordingly, AA-Exos accelerated wound closure in diabetic mice and improved perfusion/angiogenesis and collagen deposition. Together, these findings support AA priming as a process-oriented approach to improve both exosome quantity and functional potency for diabetic wound regeneration.

A central strength of our work is our mechanistic interpretation of the AA effect. We do not rely on the simple view that more particles mean better therapy. Exosomes originate from intraluminal vesicles formed in multivesicular bodies (MVBs) and are released upon fusion of mature MVBs with the plasma membrane [7,23,24]. Therefore, enhanced extracellular particles can arise from distinct biological causes, including altered ILV biogenesis, altered MVB trafficking/degradation, or altered exocytosis. Our results point to a two-step mechanism. AA raised CHMP1B, which promoted ILV biogenesis. It also raised Rab27b, which facilitated exosome release. CHMP1B knockdown lowered intracellular ILV-related signals, consistent with impaired ILV formation. Rab27b knockdown instead produced MVB accumulation inside the cell, with impaired release. This separation between biogenesis and release strengthens causal interpretation. It also addresses a recurring concern among EV reviewers. Higher yield may reflect genuine secretion capacity, or it may arise from vesicle heterogeneity and uptake artefacts. Our experiments allow these alternatives to be distinguished [14,19,27].

In parallel with the secretion-pathway interpretation, we intentionally verified that AA priming does not overtly alter fundamental exosome identity. The preservation of canonical exosomal marker proteins and the negligible detection of the negative control support that AA did not broadly convert preparations into non-exosomal vesicle populations [27]. This point is scientifically important because priming strategies that increase extracellular vesicles can be questioned if they substantially change vesicle composition independent of the intended biology. By demonstrating preserved identity alongside secretion-pathway remodeling, our results provide a more credible basis for attributing the functional differences to cargo remodeling rather than generalized EV contamination or phenotype drift.

Beyond yield, the pro-healing effect of AA-Exos is reinforced by cargo-level mechanistic evidence. Proteomic analysis revealed enrichment of pathways associated with PI3K–AKT signaling and cell adhesion molecules, consistent with a coherent endothelial repair program requiring both survival signaling and adhesion-dependent migration. Among the candidates, IL-8 emerged as a pivotal mediator: AA-Exos contained higher IL-8 levels, IL-8–FAK/AKT/ERK phosphorylation was increased after treatment, and IL-8 neutralization significantly attenuated these signaling shifts and the associated pro-repair phenotypes in endothelial cells. This interpretation aligns with the broader field in which IL-8/CXCL8 signaling regulates endothelial behavior and integrates with focal adhesion and MAPK/PI3K pathways to control motility and survival [28,29,30]. Importantly, our data suggest that the therapy is not driven by a single protein alone; adhesion/ECM-associated factors are also enriched, indicating that IL-8 likely operates within a network that coordinates receptor signaling with matrix-compatible migration dynamics. This network view is particularly relevant to diabetic wound microenvironments, where endothelial migration, survival, and matrix organization are functionally coupled [16,31,32].

The in vivo outcomes further integrate the endothelial mechanism with tissue-level repair. Diabetic wound healing is characterized by impaired microcirculation, defective angiogenesis, altered inflammatory responses, and abnormal collagen deposition. AA-Exos improved wound closure kinetics and perfusion/angiogenesis markers (VEGFA and CD31) while enhancing collagen deposition and organization. Mechanistically, improved endothelial migration/proliferation and reduced apoptosis under hyperglycemic stress provide a plausible upstream driver for improved vascularization, which then supports fibroblast-driven matrix remodeling and collagen maturation. Although multiple cell populations participate in wound repair, our study emphasizes a recipient-cell-centered mechanism: ADSC-derived exosomes reprogram endothelial signaling toward an angiogenic and pro-survival phenotype, thereby enabling downstream tissue regeneration. This interpretation is consistent with prior MSC-exosome studies demonstrating that exosomes can engage key stages of wound healing and modulate vascularization and inflammation [1].

Another notable contribution of this study is its positioning within the broader donor-priming literature. Prior work has shown that priming can enhance exosome production and therapeutic potency by reshaping secretion machinery and cargo sorting, including approaches driven by biomaterials [25] and pathway agonists [26]. Our AA-based strategy is complementary but mechanistically distinct: whereas Bioglass ion products emphasize nSMase/Rab27a-related mechanisms and miRNA cargo changes, and Wnt/β-catenin activation can promote SNAP25-dependent exocytosis and wound-healing protein enrichment, our results converge on CHMP1B–Rab27b-associated MVB/ILV and release regulation and identify IL-8–FAK/AKT/ERK signaling as the central endothelial mediator. This convergence across donor-priming modalities supports a general principle for EV manufacturing: process control should target upstream secretion/cargo pathways to improve batch consistency, rather than relying only on downstream isolation optimization [18,33,34].

Despite these strengths, several limitations should be explicitly addressed. First, we quantified exosome yield using cumulative acetylcholinesterase (AChE) activity. While AChE-based assays are widely used as operational proxies, evidence indicates that AChE is not a universal EV marker and can be sensitive to experimental context; thus, yield measurements should be interpreted as relative indicators and supported by orthogonal quantification [15,29]. Although we used particle-counting platforms and maintained identity markers, further EV purity stratification and additional release metrics (e.g., particle-to-protein normalization across preparation conditions) would strengthen the quantification framework. Second, although IL-8 neutralization attenuated AA-Exos effects, the dependence appears at least partly IL-8–mediated, implying additional cargo components contribute synergistically. Our proteomics data support enrichment of adhesion/ECM-related factors, suggesting a multi-factor cargo network. Future studies should validate additional candidate proteins (and relevant upstream regulators) using targeted loss/gain-of-function and pathway epistasis to map how IL-8 collaborates with adhesion/ECM signaling in endothelial cells under hyperglycemic stress. Third, generalizability remains to be tested. ADSCs derived from different donors, passage numbers, and culture conditions can show differences in EV secretion and cargo loading [11,16,18]. Although AA priming demonstrated consistent functional benefits in our in vitro and in vivo settings, broader validation across multiple donor batches and additional recipient cell types would be necessary to support claims of platform-level manufacturability. Beyond increasing EV yield, clinical translation of AA-primed exosomes brings practical concerns [35]. Because AA changes both exosome quantity and cargo composition, batch consistency and a clear potency definition are needed. Residual process-related components, scalability, and safety also require attention, especially since IL-8 may recruit inflammatory cells depending on its local context [36]. More broadly, for any emerging delivery platform, targeting, functional precision, low immunogenicity, and manufacturability are key [37,38].

## 4. Materials and Methods

### 4.1. Cell Culture

All experimental procedures were approved by the Ethics Committee of the Second Affiliated Hospital of Anhui Medical University. ADSCs were isolated from surgically discarded human adipose tissue from 10 independent donors (8 female and 2 male), aged 20–50 years. Donors with diabetes or hypertension were excluded. Cells at passages 3–7 (P3–P7) were used in all experiments. Briefly, minced adipose tissue was digested with type I collagenase solution (Beyotime, Shanghai, China) with constant shaking at 37 °C for 40 min until no visible tissue particles remained. The digest was centrifuged at 1000 rpm for 5 min, and the cell pellet was resuspended in DMEM/F12 medium (Gibco, Waltham, MA, USA) supplemented with 10% fetal bovine serum (FBS). The adipogenic, osteogenic and chondrogenic differentiation potential of ADSCs was verified using commercial differentiation media (OriCell) as previously described [39]. HUVECs were obtained from the Cell Bank of the Chinese Academy of Sciences (Shanghai, China). For the high-glucose (HG) condition, the glucose concentration of the culture medium was adjusted to 25 mmol/L.

### 4.2. Interference (RNAi)

RNA interference was performed by siRNA transfection using the jetPRIME^®^ transfection reagent (Polyplus-transfection S.A., Illkirch, France), following the manufacturer’s protocol. For target knockdown, three independent siRNA duplexes targeting CHMP1B and RAB27B were employed (sense/antisense sequences are provided in Supplementary Table S1). A non-targeting scrambled siRNA (NC) was used as the negative control (sequences in Supplementary Table S1).

### 4.3. Isolation and Identification of Exosomes

ADSCs were pretreated with 40 μmol/L AA in serum-free medium (Basalmedia, Shanghai, China) for 48 h to promote exosome secretion. ADSC morphology was examined under an Olympus inverted microscope (Olympus Corporation, Tokyo, Japan) at ×100 magnification using phase-contrast optics. Cell culture supernatants were collected and subjected to sequential differential centrifugation at 1000× *g* for 15 min, 2000× *g* for 15 min, and 10,000× *g* for 30 min to remove intact cells, cell debris and large microvesicles. The supernatants were then filtered through a 0.22 μm membrane to eliminate residual microparticles, followed by ultracentrifugation at 100,000× *g* for 70 min to pellet crude exosomes. The pellets were resuspended in phosphate-buffered saline (PBS; Gibco, USA) and ultracentrifuged again at 100,000× *g* for 70 min for further purification. The final pellet contained purified exosomes.

For exosome characterization, nanoparticle tracking analysis (NTA; NanoFCM, Nottingham, UK) was performed to determine the size distribution, and transmission electron microscopy (TEM; Hitachi, Tokyo, Japan) was applied to observe morphological features. Exosomal marker proteins CD9, HSP70 and TSG101 (Affinity, Changzhou, China), together with the negative control marker calnexin (Abcam, Cambridge, UK), were detected by Western blotting.

### 4.4. Acetylcholinesterase (AChE) Enzyme-Linked Immunosorbent Assay (ELISA)

The concentration of AChE in the culture supernatant of ADSCs was quantified using a commercial AChE ELISA kit (MLbio, Shanghai, China). All experimental procedures were strictly performed in accordance with the manufacturer’s instructions. The obtained AChE levels were normalized for subsequent quantitative analysis. Exosome abundance was normalized to total cellular protein content to account for differences in cell mass.

### 4.5. Exosome Uptake

ADSC-Exos were pre-labeled with the red fluorescent dye PKH26 (Sigma, St. Louis, MO, USA) and co-incubated with HUVECs for 24 h. After two washes with PBS, cells were fixed with 4% paraformaldehyde for 20 min and stained with FITC-conjugated phalloidin (Solarbio, Beijing, China) for 1 h. Cell nuclei were counterstained with Hoechst 33342 (RIBOBIO, Guangzhou, China) for 20 min. The fluorescence signals were visualized under a confocal microscope (Zeiss, Oberkochen, Germany).

### 4.6. EdU Incorporation Assay

The proliferative capacity of HUVECs was determined via an EdU incorporation assay using a commercial kit (Beyotime, China). HUVECs were seeded in 96-well plates and treated with HG, HG + Ctrl-Exos (50 μg/mL), HG + AA-Exos (50 μg/mL), or HG + AA-Exos + Anti-IL-8 (5 μg/mL, Abcam). After the corresponding treatment, the culture medium was replaced with EdU-containing medium and cells were incubated for 2 h. Subsequent operational steps should be followed according to the instructions provided in the BeyoClick™ Plus EdU-488 Cell Proliferation Detection Kit manual. Then stained cells were visualized and imaged under a microscope (Zeiss, Germany).

### 4.7. Cell Cycle Analysis

Flow cytometry was applied to evaluate the cell-cycle profile of HUVECs using a commercially available cell cycle detection kit (Abbkine, Atlanta, GA, USA). Following the indicated treatments, cells were harvested by trypsinization, washed twice with PBS, and centrifuged to collect cell pellets. Cells were then fixed with pre-chilled 70% ethanol at 4 °C overnight. After fixation, cells were washed with PBS again and resuspended in staining solution according to the manufacturer’s protocol. Subsequently, cells were incubated with propidium iodide (PI) in the dark at room temperature for 30 min. Cell cycle profiles were acquired and analyzed on a flow cytometer (Beckman, Brea, CA, USA).

### 4.8. Cell Apoptosis Assay

Cells in the logarithmic growth phase were collected and adjusted to a density of 1 × 10^6^ cells/mL, followed by centrifugation to remove the supernatant. After two washes with pre-chilled PBS, the cell pellet was resuspended in 500 μL of binding buffer, after which 5 μL of Annexin V-FITC and 5 μL of propidium iodide (PI) were added sequentially. Following incubation of the cell suspension at room temperature in darkness for 15 min, flow cytometric analysis was completed within 1 h. Early apoptotic cells were defined as the Annexin V^+^/PI^−^ population, and late apoptotic cells were defined as the Annexin V^+^/PI^+^ population.

### 4.9. Tube Formation Assay

Tube formation capacity was assessed using Matrigel (Corning, NY, USA). Matrigel was diluted with DMEM at a 1:1 ratio and added to pre-chilled 24-well plates at 200 μL per well, with all operations performed on ice. HUVECs receiving different treatments were seeded onto Matrigel-coated plates at a density of 5 × 10^4^ cells per well and incubated at 37 °C for 6 h. Capillary-like structures were visualized under an microscope (Zeiss, Germany), and the number of formed tubes was quantified by Fiji (ImageJ) version 2.0.

### 4.10. Wound Healing Assay

HUVECs were seeded in 6-well plates. At 90% confluence, a uniform scratch was created per well using a sterile 200 μL pipette tip. After washing twice with PBS to remove floating cells, cells were subjected to different treatments. Scratch images were acquired at 0, 24, and 48 h under an optical microscope (Olympus, Japan), and the wound closure percentage was quantified using ImageJ software.

### 4.11. Transwell Migration Assay

Cell migratory capacity was evaluated using 24-well Transwell chambers with 8-μm pore size membranes. Briefly, HUVECs resuspended in serum-free medium were seeded into the upper chambers, while medium containing exosomes or corresponding reagents was added to the lower chambers. After incubation, the membranes were fixed with 4% paraformaldehyde at room temperature and stained with crystal violet. Migrated cells on the lower membrane surface were observed using a microscope (Zeiss, Germany).

### 4.12. Western Blot Analysis

Proteins were separated by sodium dodecyl sulfate-polyacrylamide gel electrophoresis (SDS-PAGE) and electrotransferred onto polyvinylidene difluoride (PVDF) membranes (Millipore, Burlington, MA, USA). Membranes were blocked with 5% bovine serum albumin (BSA), incubated with primary antibodies overnight at 4 °C, and washed with TBST. Thereafter, membranes were incubated with horseradish peroxidase (HRP)-conjugated secondary antibodies for 1 h at room temperature. Protein bands were visualized using enhanced chemiluminescence (ECL) reagent (Abbkine, USA) with a chemiluminescence imaging system (Bltlux, Guangzhou,China ). Band intensity was quantified via ImageJ software and normalized to the loading control β-actin.

The primary antibodies used were as follows: Akt, phosphorylated Akt (p-Akt), FAK, phosphorylated FAK (p-FAK), ERK, phosphorylated ERK (p-ERK) (Affinity, China); β-actin (Proteintech, Wuhan, China); calnexin, CD9, TSG101, HSP70 (Abcam, UK).

### 4.13. Transcriptomic Analysis

ADSCs were divided into two groups, including a control group (Ctrl) and an AA-treated group, with three biological replicates per group. After routine culture under specified conditions, total RNA was isolated from cell samples using the Trizol reagent. All RNA samples were treated with DNase I and purified via magnetic beads to eliminate genomic DNA contamination. Only samples with an RNA concentration above 200 ng/μL and an RNA integrity number (RIN) ≥ 8.0 were qualified for subsequent library construction. Messenger RNA (mRNA) was enriched using Oligo(dT) magnetic beads and fragmented randomly for cDNA library preparation. The quality of the constructed libraries was validated using an Agilent 5400 Bioanalyzer (Agilent Technologies, Santa Clara, CA, USA) prior to high-throughput sequencing on the BGI Illumina platform. Raw sequencing reads were quality-controlled with fastp (v0.23.4) to remove low-quality sequences. Gene expression levels were quantified in transcripts per million (TPM) using the StringTie2 (v2.1.4). Differentially expressed genes (DEGs) were identified using DESeq2(v1.38.3) with thresholds of |log2 fold change| > 1.0 and adjusted *p* value (Benjamini–Hochberg FDR) < 0.05. Gene Ontology (GO) and Kyoto Encyclopedia of Genes and Genomes (KEGG) enrichment analyses were further performed to explore the functional characteristics of the identified DEGs.

### 4.14. Exosomal Proteomic Analysis

Exosomes derived from ADSCs were divided into two groups, namely the Ctrl-Exos group and AA-Exos group, with three biological replicates in each group. After cell culture under defined conditions, exosome samples from each group were lysed with 120 μL of RIPA lysis buffer. Ultrasonic lysis was performed on ice for 20 min using a cell disruptor at 12,000 rpm. The lysates were centrifuged at 4 °C for 10 min, and the resulting supernatants were collected for total protein quantification using the BCA assay. Subsequently, protein reduction, alkylation, and enzymatic hydrolysis were performed for peptide preparation. Equal amounts of peptides (500 ng per sample) were separated using a Vanquish neo nano-UPLC system (Thermo Fisher Scientific, Waltham, MA, USA), followed by mass spectrometry data acquisition using a Thermo Scientific Astral mass spectrometer (Thermo Fisher Scientific, Waltham, MA, USA) with a nanoelectrospray ionization source. All samples were analyzed in data-independent acquisition (DIA) mode against the target sequence database. DIA-based proteomic analysis of exosomes was performed using DIA-NN (version 2.2.0) for database searching against the human UniProt reference proteome (9606_all.fasta). Digestion was carried out with trypsin allowing up to two missed cleavages; carbamidomethylation of cysteine was set as a fixed modification, and acetylation (N-terminus) and oxidation of methionine as variable modifications. Peptide identification was controlled at a false discovery rate (FDR) of 1%. Missing quantitative values were filled with half of the minimum value of the quantitative data. Differentially expressed proteins were identified by Student’s *t*-test with thresholds of fold change < 0.83 or >1.2 and *p* < 0.05, and Benjamini–Hochberg-adjusted *p* values are reported in the Supplementary Excel S2.

### 4.15. In Vivo Wound Healing Assay

Male db/db mice aged 6–8 weeks were used for wound healing evaluation. Anesthesia was induced via intraperitoneal injection of 1.25% Avertin solution (0.2 mL per 10 g body weight; Easycheck). Dorsal hair was first clipped and then removed with depilatory cream, followed by rinsing with water. Mice were randomly divided into three groups (n = 7 per group) and injected with 200 μL PBS, 200 μg Ctrl-Exos (resuspended in 200 μL PBS), or 200 μg AA-Exos (resuspended in 200 μL PBS), respectively. Wound images were captured on days 0, 3, 7, 10, and 14 post-surgery, and wound closure was quantified by ImageJ software. Mice were euthanized on day 14, and wound tissue samples were collected for subsequent histological section staining and immunohistochemical analysis.

### 4.16. Laser Speckle Flow Imaging

Laser speckle flow imaging was performed using the Moor O2FLO-2 tissue blood flow imaging system (Moor Instruments, Axminster, UK) to acquire dynamic visual maps of tissue perfusion around mouse wounds. Fully expose the mouse wound area for scanning probe microscopy. The scanning probe was positioned at a 15° perpendicular angle against the tissue surface, with the scanning window maintained 10–38 cm away from the measurement site. Mean perfusion values were quantified within the wound bed (ROI1) and at a standardized distance from the wound margin (ROI2). The average perfusion ratio was calculated by comparing ROI1 to ROI2, and corresponding blood flow images were saved. Raw data were processed via moorO2FLO-2 Image Review V1.0, followed by statistical analysis in GraphPad software 10.1.2.

### 4.17. Histological Analysis

Excised wound tissues were fixed in 4% paraformaldehyde, dehydrated, and paraffin-embedded, followed by tissue sectioning. Sections were dewaxed and subjected to hematoxylin-eosin (H&E) and Masson’s trichrome (MT) staining to assess re-epithelialization and collagen maturation, respectively. Stained sections were visualized under an optical microscope. For immunohistochemistry, paraffin sections were rehydrated and incubated with primary antibodies targeting VEGF and CD31, followed by secondary antibody incubation. Immunoreactivity was detected using a DAB chromogenic substrate under optical microscopy. Sirius Red-stained sections were imaged under polarized light to quantify the relative abundance of collagen I and collagen III.

### 4.18. Statistical Analysis

All quantitative data were obtained from no fewer than three independent biological experiments and are presented as mean ± standard deviation (SD). Statistical analyses were performed using GraphPad Prism 10.1.2 (GraphPad Software, San Diego, CA, USA). Student’s *t*-test was used for comparisons between two groups, and one-way analysis of variance (ANOVA) followed by Tukey’s post-hoc test was applied for multi-group comparisons. For time-course data, including cumulative AChE levels and wound closure rates, two-way repeated-measures ANOVA with treatment and time as experimental factors was used, followed by Tukey’s test (three groups) or Šidák’s test (two groups) for multiple comparisons. A *p*-value < 0.05 was considered statistically significant.

## 5. Conclusions

In this study, we demonstrated that arachidonic acid (AA) pretreatment of adipose-derived stem cells markedly enhances exosome production without altering exosome identity or fundamental properties. Mechanistically, AA increased the expression of the MVB/ILV biogenesis and exocytosis regulators CHMP1B and Rab27b, thereby promoting MVB/ILV formation and facilitating the release of mature exosomes. Importantly, AA also remodeled the exosomal cargo: exosomal proteomics and mechanistic validation identified IL-8 (CXCL8) and cell adhesion/ECM-related factors as key mediators, with AA-Exos activating the IL-8–FAK/AKT/ERK signaling axis. Functionally, AA-Exos displayed superior therapeutic efficacy in vivo, significantly accelerating diabetic wound closure, improving blood perfusion and angiogenesis (including VEGFA and CD31), and enhancing collagen deposition and organization. Consistently, AA-Exos promoted high-glucose–challenged endothelial cell migration and proliferation while inhibiting apoptosis, and these benefits were at least partly dependent on IL-8 signaling. Collectively, AA-priming offers a practical and mechanistically supported strategy to generate standardized, high-yield, pro-healing ADSC-derived exosomes, advancing their translational potential for diabetic wound regeneration.

## Data Availability

The original contributions presented in this study are included in the article/Supplementary Materials. Further inquiries can be directed to the corresponding author.

## Supplementary Materials

The following supporting information can be downloaded at: https://www.mdpi.com/article/10.3390/ijms27209029/s1.

## Abbreviations

The following abbreviations are used in this manuscript:

ADSCs	adipose-derived stem cells
CTRL-Exos	Control exosomes
AA-Exos	exosomes from ADSCs pretreated with arachidonic acid
AA	arachidonic acid
HUVECs	human umbilical vein endothelial cells
HG	high-glucose
VEGF	Vascular Endothelial Growth Factor
MSCs	mesenchymal stem cells
Exo	Exosome
EVs	extracellular vesicles
TEM	Transmission electron microscopy
NTA	Nanoparticle tracking analysis
KEGG	Kyoto Encyclopedia of Genes and Genomes
GO	Gene Ontology
DM	diabetes mellitus
MVB	Multivesicular bodies
ILV	Intraluminal vesicles
